# “In the driver’s seat”: The Health Sector Strategic Master Plan as an instrument for aid coordination in Mongolia

**DOI:** 10.1186/1744-8603-10-23

**Published:** 2014-04-03

**Authors:** Anar Ulikpan, Indermohan Narula, Asmat Malik, Peter Hill

**Affiliations:** 1 School of Population Health, The University of Queensland, Herston Road, QLD-4006 Herston, Australia; 2 Ministry of Health Mongolia, Government Building 8, Olympic Street 2, 14210 Sukhbaatar District, Mongolia; 3 Integrated Health Services, House 6, street 48, f-8/4, Islamabad, Pakistan

## Abstract

In 2005, the Ministry of Health (MoH) in Mongolia initiated the process of developing its Health Sector Strategic Master Plan (HSSMP), using a wide-ranging consultative process, driven by the MoH, and requiring participation from all levels of health facilities, other ministries, donor agencies and NGOs. Among other objectives, the MoH sought to coordinate the disparate inputs from key donors through the HSSMP, aligning them with the Plan’s structure. This research explores the extent to which the HSSMP process served as a mechanism for effective aid coordination while promoting ownership and capacity building and the lessons learned for the wider international development community. The study is based on document review, key-informant interviews and authors’ experience and participation in the MoH planning processes. The HSSMP process improved alignment and harmonisation. It enabled a better local understanding of the benefits of aid coordination, and the recognition that aid coordination as not only a mere administrative task, but a strategic step towards comprehensive management of both domestic and external resources. The process was not challenge free; the fractious political environment, the frequent turnover of key MoH staff, the resistance of some donors towards MoH scrutiny over their programmes and the dismantling of the central coordination and return of seconded staff following completion of the HSSMP, has slowed the pace of reform. Despite the challenges, the approach resulted in positive outcomes in the areas of ownership and better aid coordination, with HSSMP development emphasising ownership and capacity building. This contrasted with the usual outcomes focus, and neglect of the capacity building learning processes and structural and policy changes needed to ensure sustainable change. The largest and most influential programmes in the health sector are now largely aligned with HSSMP strategies, enabling the MoH to utilize these opportunities to optimise the HSSMP outcomes. The lessons for Ministries of Health in similar Post-Soviet countries--or other emerging economies where government capacity and local policy processes are relatively strong--are clear: the development of solid governance and technical infrastructure in terms of planning and evaluation provide a solid structure for donor coordination and insure against local political change.

## Background

In 2005, the Paris Declaration on Aid Effectiveness was endorsed by more than 100 signatories—from donor and developing-country governments, multilateral donor agencies, regional development banks and international agencies—with the commitment to improve aid effectiveness and the harmonization of development [1]. The Declaration asserted partner countries’ *ownership* over their own development policies, *alignment, harmonization, results and mutual accountability* as working principles for effective aid [1]. But despite the explicit emphasis on local leadership and ownership as prerequisite conditions for aid effectiveness, in most developing countries, the development agenda is frequently driven by donors [2,3]. Global reviews of progress towards the Paris Declaration targets have also highlighted the uneven transition of ownership from donors to partner countries, and concepts of ownership are often interpreted differently by different actors [2,4]. The Accra Agenda for Action sought to address this, urging donors to promote ‘real’ country ownership [5] and ‘walk’ the talk by changing the way aid is delivered [6]. While there is a limited literature on the challenges of aid coordination from the perspectives of developing countries [7-9] no accounts of the aid transition in a post-Soviet health system has been previously documented.

This paper examines government ownership through the development of the Health Sector Strategic Master Plan (HSSMP) as a mechanism for securing donor coordination, based on documentary analysis, key informant interviews and participant observation undertaken within the Mongolian health system.

In 2005, Mongolia, recovering from major socio-economic challenges following the collapse of the Soviet Union, and still transitioning from centrally planned socialism to democracy, released its Health Sector Strategic Master Plan (HSSMP) 2006–2015 [10], asserting its own health sector policy directions. The decision was crucial for the development of the health sector, marking a definitive shift in Ministry of Health (MoH) relationships with donors. Prior to the collapse of the Soviet Union in 1991, Mongolia was dependent solely on the Soviet Union for aid, and had no previous experience working with other donors. To adapt to the democratic transition, Mongolia needed to embark upon reforms in all sectors requiring support from a new range of donors. With the breakdown of the Soviet system, five-year Soviet type plans were discontinued, without any compensatory comprehensive long-term planning mechanism in place for the health sector from 1991 to 2005. In this vacuum, development objectives were determined largely by donors, with development assistance delivered mainly as projects, fragmenting an already fragile health system—still strongly centralized and hospital based following the Semashko model [10]. The Semashko model was established in the 1920s and operated throughout the Soviet Union until early 1990s [11]. “*The model was characterized by its centralized planning and administration, government financing and provision of services through publicly owned health care providers, which were universally accessible and free at the point of delivery*” (p. 421) [11]. However, with the collapse of the Soviet Union it was too costly to maintain the model as it is considered “inappropriate and inefficient” to meet the changing health needs of the population.

Support from donors between 1991 and 2003 averaged 40% of GDP [12,13]. The multilateral agencies (United Nations Children’s Fund (UNICEF), United Nations Population Fund (UNFPA), World Health Organization (WHO) and Asian Development Bank (ADB) along with bilateral partners (Japanese International Cooperation Agency (JICA), German Agency for Development Cooperation (GTZ) (now renamed as German Agency for International Cooperation (GIZ), the European Union) and some international Non-Government Organizations (World Vision, Voluntary Service Overseas) played key roles in health, but using a disparate range of approaches and objectives. Total health sector expenditure over the study period and contributions of the key donors in health and their contributory areas have been provided in Additional file 1 to allow readers to have a better understanding of the aid provided in the Mongolian health sector.

Before 2003, coordination of donors and external resources by the MoH was very fragmented. There was no sector-wide coordinating mechanism within the MoH to provide a consultative forum involving the various departments of the MoH, donors, NGOs and beneficiaries. Different MoH departments presented their perspectives and priorities directly to donors, resulting in duplication of projects being implemented, and the formation of multiple Project Implementation Units and parallel management systems [14]. Project proposals were designed by donors for the MoH’s approval, and were often approved without critical consideration of their relevance and appropriateness, given the government’s chronic funding shortages and imprecise sector priorities [14]. Projects were managed independently by their Project Implementation Units, and were insulated from the rest of the system because of agency accountability requirements. The demand for project management staff diverted limited human resources from the MoH to serve project interests.

Despite the benefits of development assistance, the systemic costs were becoming increasingly evident. Poor information sharing and feedback between the projects, donors, the MoH and beneficiaries highlighted an urgent need for a sectoral approach in planning, resource mobilization and coordination [15]. The MoH recognized that effective coordination—of its own departments as well as the international donors and agencies supporting these disparate initiatives—was a necessary mechanism to promote its health system reforms, and that a strategic sectoral planning process was an appropriate mechanism for achieving this. While the development literature is rich in its rhetoric about local ownership in health, there are limited examples of how putting the government “in the driver’s seat”— has been successfully achieved. In Mozambique, re-orientation of the aid coordination mechanism under government leadership revealed a lack of government capacity to manage the coordination of resources [7]. In the case of Cambodia, despite the growing interest within the Government to facilitate sector-wide management, limited MoH capacity necessitated the extensive participation of WHO and other consultants in the early phases of the reforms [8]. The excessive influence of donors on Ugandan health policy development, potentially threatened national sovereignty and the sustainability of the policy [9]. Having suffered seven decades of Soviet dominance, the Mongolian government was eager to learn from these experiences. This research case-study documents the HSSMP process, specifically examining the ways in which it was used as a mechanism to build effective aid coordination, while nurturing local ownership and enabling capacity building in planning and management, and the challenges that implementation now faces.

## Methods

The research uses a health systems case-study approach, examining the evolution of the HSSMP over the decade beginning from 2003, principally using qualitative methods: document review of peer-reviewed journal articles, unpublished studies, government policy and program documents, international agency and institutional reports, progress reports on the implementation of the Paris Principles in Mongolia; semi-structured interviews with 23 key informants, purposively selected to inform on the early and mid-implementation phases of the HSSMP; participant observation of key events [16] by the authors (AU, IN) including participation in implementing health reforms from 2003 to 2010; engaging in preliminary strategic planning, HSSMP development and its implementation processes; the formation of aid coordination committees, and experience of the changes in structure and function of aid coordination responsibilities within the MoH. Rigor within the study was enhanced by triangulation of findings from the three approaches and inclusion of authors with familiarity with the Mongolian health system, but external to the MoH [17].

The Key Informant Interviews were undertaken in 2008 (12 participants), in the early stage of the HSSMP implementation [18] and again in 2012 (11 participants). This allowed researchers to track progress from the strategic planning stage through to implementation and to observe perceptions and paradigm shifts over time, as the dominance of the health sector planning agenda shifted from donors to the MoH. Both sets of interviews included equal representatives of key partners in the health sector: bilateral and multilateral institutions, development banks, international NGOs and government staff working at central and aimag (province) levels.

## Findings and discussion

The research focuses on two phases of the HSSMP process: the *development* of the plan and its implementation framework (2003–2006) and its subsequent *implementation* (2006–2012). The HSSMP development phase was preceded by MoH’s recognition of the need for a strategic direction and coordination of resources using a sectoral planning process, and its commitment to ownership through a ‘unique’ team arrangement. This arrangement differed from previous Project Implementation Units by being located centrally within the MoH, and relying on high levels of MoH staff participation. A participatory situation analysis undertaken by the MoH with international partners was the first challenging step of the HSSMP development process. This collaborative review exposed the reality on the ground of the MoH’s own health system to the scrutiny of donors and other domestic and international stakeholders.

The HSSMP development phase was characterized by three distinct features:

1. process orientation instead of a focus on quick results

2. an implementation framework developed concurrently for the training of the responsible implementers, to ensure capacity building for smooth implementation

3. active management of key donors and development partners through the HSSMP process.

HSSMP implementation reinforced the ownership derived from the HSSMP process, leading to continued commitment of the MoH and the main international partners to the HSSMP. It enabled a better understanding of the benefits of aid coordination, which brought about a “paradigm shift” within the MoH that reframed aid coordination as not only a mere administrative task, but a strategic step towards comprehensive management of both domestic and external resources. The challenges faced during the HSSMP implementation provided lessons learned for future reform processes.

### HSSMP development

#### Commitment to ownership: the ‘unique’ project team arrangement

In 2001, the MoH Secretary of State and senior bureaucrats took the initiative to begin a strategic planning process. Over a two-year period (2001–2003), a dialogue between the Ministers of Health of Mongolia and Japan established an agreement on the approaches and arrangements for technical assistance for HSSMP development. The choice of partner in this process engaged a strategic regional partner, bypassing other Western bilateral partners with a higher profile interest in health sector reform at the time. Although Japan was Mongolia’s largest current donor, capacity building initiatives that granted ownership to the recipient country were not common in their development assistance practice. Despite this, the Mongolian MoH was able to persuade its counterpart to offer a flexible approach through the Japanese International Corporation for Welfare Services (JICWELS), an implementing agency of the Japanese MoH, that was supportive of capacity building and ownership [19].

**Figure 1:**
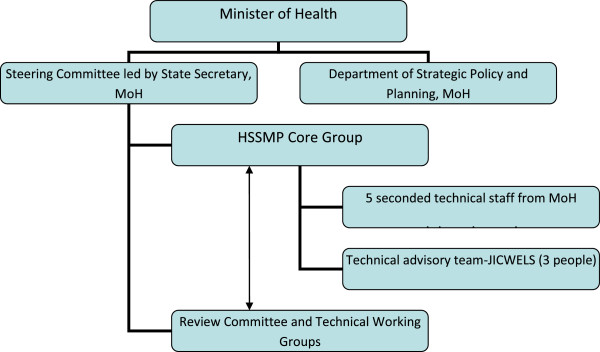
Organizational structure of the HSSMP initiative.

Instead of the typical Project Implementation Unit (PIU), insulating project staff and its operations from the MoH, the MoH formed a HSSMP Core Group consisting of 5 technical staff seconded from the MoH and a small JICWELS technical advisory team of three staff (a long-term Technical Advisor, and Technical and Logistics Officers). This was embedded within the MoH structure, with a counterpart relationship with the Department of Strategic Policy and Planning of the MoH, and reporting to a Steering Committee led by the State Secretary, MoH (Figure 1). The functional nature of this arrangement enabled the integration of the initiative into the planning functions of the MoH, contributing to ownership, capacity building and sustainability within the MoH.

Technical Working Groups (TWGs) were established through ministerial orders to develop strategies for priority areas, which were identified during the situation analysis. The ministerial orders mandated the participation of key senior and mid-level staff in the TWGs in the development of the HSSMP with representation from service delivery facilities, academia, donors and NGOs, establishing the basis for the coordination of partner inputs from the onset of the initiative. The Core Group, in consultation with the key MoH staff, developed a roadmap (Additional file 2) before setting up the TWGs. This roadmap was discussed and endorsed by the key donors allowing the process to be open and transparent from the beginning, but the structure ensured ownership was maintained within the Core Group without being dominated by the donors, with donors invited to participate as members of selected TWGs based on their technical expertise.

#### The situation analysis

The planning process began with a comprehensive situational analysis of the health sector involving both local planners and international actors (multi-laterals, development banks, bilateral donors and NGOs). The review was based on an extensive review of the 192 available reports by consultants and government, grey literature and research findings produced over the 5 years prior the HSSMP process.

The situation analysis was undertaken by the Core Group as the first task of the HSSMP development process overseen by the Steering Committee and supported by the local decision makers and donors in health. Senior MoH management were concerned that without such an analysis there was the risk that the planning process would lead to the reinforcement of existing Semashko model based policies, now recognized as inadequate in responding to the sector’s needs. The analysis further reinforced the need for sectoral reform, and the need to build the capacity of the local leadership, if the MoH, rather than donors, was to retain ownership of the process [14]. However, the process was not challenge free. The MoH commitment to transparency in assessing its own system in collaboration with donors and other stakeholders, pointed to their own weaknesses, while simultaneously identifying the need to move towards a better functioning health system, responsive to the changing socio-economic, demographic and epidemiological circumstances.

The MoH then took primary responsibility for using the development of the HSSMP as the mechanism for building health sector capacity in close collaboration with other ministries and donors, with support from JICWELS. [20]. Offers from a key international partner to provide external consultants to draft the HSSMP on behalf of the MoH were declined, despite the promise that this might make the HSSMP more acceptable to broader donors. This courage to reject partners’ offer resulted from previous experience-failed reform initiatives driven by external consultants. Examples of these reforms are decentralisation and health sector privatisation, which were instituted during 1993–1996 along with the introduction of the Public Sector Management and Finance Law (a modified version of Australian Public Sector Management Act 1994) and the implementation of the Health Sector Development Programme-1 by ADB. While technically these reforms addressed issues of governance and significant public policies, the failure of the consultants to understand the politics produced by the rapid transition from a central control economy to more democratic institutions meant that the necessary local policy ownership was not achieved, and the regulatory changes needed for both reforms were not implemented. Progressive undermining within the administration over several years, and frequent changes in government, resulted in the failure to implement these reforms. The key reasons for the failure were defined as a lack of prior preparation, the absence of well-defined and harmonised guidelines and implementation mechanisms, and inadequate systematic training of the managers at the local government level [14]. In the light of this experience, the MoH aimed to own the process through to implementation, engaging local health planners and allowing them to “learn by doing”. Despite some donor ambivalence around the MoH staff’s capacity to manage this process, support was maintained during this phase. For the MoH, the assertion of leadership enabled a change in their own practices: without externally imposed time constraints or donor conditionalities, the MoH was able to place an equal emphasis on the process as well as the results. This was a significant development, as process-orientation and taking ownership in its relationship with the international partners had not been part of the MoH organizational culture, as underscored by many interviewees representing Government agencies: *“Traditionally, international partners initiated project planning and set up their own project management and coordinating mechanisms and MoH followed their arrangements. But HSSMP process was different; it switched the “seats”. The Government took a “driving seat” for the first time…”* (Senior MoH official).

The process also promoted participation of various actors such as health workers in bagh and soum (peripheral administrative units), aimag, NGOs, other sectors’ representatives and private practitioners, welcoming the fresh inputs and perspectives from these heterogeneous actors.

### HSSMP implementation

#### Implementing the plan and training the implementers

The concomitant development of the three companion documents of the HSSMP—the Planning and Budgeting, Medium-Term Expenditure and Monitoring and Evaluation Frameworks, supplemented by an Implementation Framework—served as the apparatus for the actual implementation of the Plan at the operational levels. The Implementation Framework formed the basis for the Government’s Action Plan in Health and the Mid-term Plan of the MoH. These companion frameworks provided the necessary guidelines, forms checklists and tools for preparing facility level annual operational plans. Preparation of these plans was managed and facilitated by the MoH, with assistance from the HSSMP Core Group, through a series of participatory training events covering all regions. This enabled the aimag and district facility management teams to develop integrated annual operational plans for all health facilities at each level. These events used a “learning by doing” approach to build the capacity of the health management teams in planning, budgeting, monitoring and evaluation. During the training events, participants recognized that up to this point, the annual planning and budget estimation had not been linked, and that for effective planning, this linkage was vital. Ongoing in-service training, provided by the MoH, would be required if the emerging ability to plan, estimate budgets, implement and monitor the annual work plans was to be institutionalized.

The Implementation Framework was an essential tool to help unpack national level strategies into implementable objectives and activities that could be adapted at the aimag and soum (district) level, while the participatory training methodologies equipped the health management teams with the necessary skills to develop their operational plans and budget estimates. Despite the MoH’s reticence to delegate control of the planning process to donors, the need for donor support for implementation was increasingly self-evident. For the donors, the functional structure emerging from the planning process raised confidence in the HSSMP. Consultation to secure the support of ADB, WHO, GTZ, UNICEF and UNFPA, made the strategic plan amenable for implementation at operational levels. These partners now also adapted their own strategic plans to reflect the HSSMP strategies, providing funding for training in their programme area health facilities at aimag and soum levels.

During the development and implementation process, three national consultative meetings and 16 regional and aimag level consultative meetings were held. These provided additional capacity building opportunities to examine local and sector-wide priority issues and make recommendations. These meetings also enabled consensus building about these priorities and suitable implementation modalities. A number of interviewees from implementation levels positively commented on the ownership aspect of the plan. Their views are represented in the following quote from a Senior health official of the Aimag Health Department *“This was the first time the implementation plans were developed by us, the implementers, and not just imposed on us by outsiders or top level MoH and related government agencies as happened often in the past”.* Although institutionalizing the planning exercise at the operational level was constrained by the lack of capacity, experience and resources, the shift in mindset brought about by this planning process was significant: the assertion of ownership of the process by the MoH now enabled the evolution of local ownership by aimag and city health departments of these operational plans.

#### Managing donor participation

With the cabinet approval of the sector strategic plan in 2005, coordination of donors under the MoH leadership became necessary to enable MoH to begin managing the sector. The recent declaration of the Paris Principles provided further impetus for harmonisation of donor planning with that of the MoH. The increasing focus on Health Systems Strengthening as a global shift in development assistance saw some key partners (GTZ, UNFPA, UNICEF) providing funds for HSSMP supported training activities for MoH staff in their project areas. In the health system strengthening components of their plans, this provided evidence of their buy-in into MoH capacity building. Broad acceptance of the importance of host country ownership and capacity building was becoming evident in action plans to direct donor coordination through a Sector-Wide Approach (SWAp) [18]. While a SWAp is conventionally understood as a donor coordination mechanism in which partners, under the leadership of the MoH, align and harmonize all resources and efforts through the collaborative development of a single sector plan [21,22], the experience in Mongolia effectively inverted this sequence. The development of the HSSMP with managed donor input, provided an initial mechanism to assert MoH ownership, and build capacity. Now the HSSMP would serve as a structure to harmonize donors’ (particularly ADB, GIZ and UNFPA) and other stakeholders’ contributions, aligning donors’ agendas with the MoH policy package, and setting the agenda for a future SWAp.

#### The HSSMP as an ongoing construct for coordination

Given the history of frequently changing priorities in the MoH with each new ministerial regime and its administration, the durability and continuity of the HSSMP had to be carefully considered from the outset.

First, wider acceptance by a broader range of stakeholders was necessary. A process of reviews to build advocacy for approval was planned: a consultative meeting involving all the directors of the aimag health departments, heads of the main tertiary hospitals and heads of the MoH departments was held to endorse and submit a communique, signed by all the participants and approved by the State Secretary, to the MoH, urging the adoption of the plan.

Next, a Review Committee was appointed consisting of senior MoH staff and key donors to ensure the consistency of plan with the MoH and partners’ priorities. The endorsement of the Cabinet was imperative. The plan was then revised and submitted to the Minister’s Council for approval, and for subsequent presentation to the other Ministries for feedback to obtain their commitment to collaborate with the MoH in implementation. This part of the review process was required for presentation of the HSSMP to the cabinet for approval. The Cabinet approved the HSSMP and its companion documents, and a Resolution endorsing the HSSMP and authorizing the Minister of Finance to fund the plan with active support from the partners, was signed by the Prime Minister of Mongolia [23]. As in other documented country experience, approval of the strategies at a level higher than the MoH were deemed to be beneficial for achieving better donor coordination and continuity [24]. This was also demonstrated by the Mongolian HSSMP process, and increased the commitment by the MoH-Mongolia to the HSSMP. The process also helped to provide legitimacy so it could continue to serve as the primary umbrella document, despite subsequent changes within the Minister of Health. Consequently, each new Minister has, until now, employed the HSSMP as the basis for developing the Ministry’s work plans.

Second, the Steering Committee appointed by the Minister to oversee the HSSMP process played a central role in safeguarding the continuation of the process of coordination and harmonization beyond the development of the HSSMP. Its members went on to serve as members of the Health Sector Aid Coordinating Committee (HSACC). The HSACC was newly established in 2005, as required by the Ministry of Finance (MoF), following the HSSMP’s approval, as a mechanism for supporting a SWAp. Regular meetings of this committee enabled future donor initiated projects to be in line with the HSSMP strategies and current programmes to be coordinated under the umbrella of HSSMP. During these meetings, progress reports of current projects and new project proposals were presented by the MoH and partners for consultation and approval. Also, project and programme evaluation reports were presented at these meetings and new studies, and initiatives such as the joint sector review were consulted upon and recommended for implementation by the MoH and partners.

The 2008 general elections, however, exposed the potential vulnerability of coordination to political change: the formation of a coalition government resulted in the health portfolio being transferred to the minority coalition partner. As a result, HSACC operations were suspended for about a year. In this interim period, however, the MoH, recognizing the importance of the coordination function, was able to continue the alignment and harmonization of projects with the HSSMP by appointing temporary technical working groups. These efforts were supported by the key international partners in the health sector.

With the support of ADB and WHO, the MoH has regularized the meetings of the HSACC and is now moving towards expanding its role as a Health Sector Coordinating Committee to further support the Health Sector Reform Agenda. Additionally, key partners now operate through the budgetary process approved by MoF. Evaluation of the Paris Declaration activities in Mongolia indicated that donor use of country public financial management systems has increased from 17% to 27% between 2007–2010, although this is still below the target set for 2010 [25].

#### Improved aid coordination

In practice, HSSMP development enabled harmonization and coordination of external aid earlier than anticipated, as the largest international partners oriented their support towards MoH priorities. Key partners in health increased their support to the health sector, and aligned these with HSSMP priorities [26]. The Third, Fourth and Fifth Health Sector Development Projects (HSDP) funded by Asian Development Bank (17.6 million; 18.15 million and 30 million USD respectively) focus on the key strategies outlined in the HSSMP: improving health insurance system, hospital rationalization, strengthening primary health care, improving postgraduate clinical training, drug safety, blood safety and waste management.

UNICEF and UNFPA budgets doubled between 2006 and 2010 [26]. After four years of inactivity, the WB program contribution to the health sector resumed in 2007. For GIZ, after an absence of 6 years, health support recommenced in 2011. Their support focused on addressing capacity building in management for emerging infectious diseases, and the introduction of social health insurance, which were listed as key strategies in the HSSMP. The US government funded (17 million USD) Millennium Challenge Account project 2008–2013 addressed health issues for the first time: the increasing threats of non-communicable disease and road traffic trauma. In interviews, the key informants from bilateral and multilateral agencies unanimously agreed that HSSMP provided a predictable structure for channeling their resources in health in accordance with MoH plans:

*“.. We are happy to work with MoH as its scope and direction is clear and priorities identified in the Ministry’s long term plan accurately pinpoints areas to be improved in Mongolian health sector…Our ultimate intention is to bring sustainability within the system which thankfully, was also key emphasis in the Ministry’s master plan. The plans often used to be merely a “wish list” in the past”.* (Multilateral donor representative)

Following HSSMP approval, unspecified donor funding for health decreased consistently from 40.3% in 2003 to 3.7% in 2007, reflecting alignment with HSSMP priorities [27]. However, alignment was clearly dependent on MoH monitoring: following the 2008 elections and the suspension of the Health Sector Aid Coordination Committee, unspecified funding increased to 19% in 2009. The total health expenditure as a percentage of GDP also increased, from 4.8% in 2006 to 5.5% in 2010 [28]. Progress towards the Millennium Development Goals for health is well on track, with the target for reducing maternal mortality met before 2015 [29,30]. Although these positive contributions may not be solely attributed to the HSSMP, its more targeted and coordinated ways of using health resources have supported development of the health sector and resultant health outcomes.

Three specific examples demonstrate the use of the HSSMP as an instrument for securing donor coordination. As a lower income country moving towards middle-income status, Mongolia continues to be eligible for grants. While the Government had not been heavily involved in the design of grant projects, they now sought to ensure that projects were aligned with HSSMP strategies. Although the original design of the THSDP was highly focused on an external consultancy model, marginalizing local engagement, the MoH insisted that this program and subsequent ADB grants now conform to the HSSMP, under the oversight of the HSACC. The three biggest ADB health projects are currently operated under a single PIU under the HSACC, allowing better harmonization and alignment between the projects and MoH, besides substantially saving management costs. All the funding provided by ADB and WB is now channeled through the Ministry of Finance, rather than off budget.

The second example involved the Millennium Challenge Account-Health project, whose conditions required a demonstrable business orientation as part of the proposal. The resultant proposal promoted the establishment of a quasi-private tertiary level diagnostic and treatment center, designed with the intention of meeting the health needs of the wealthier members of society, and capturing the health funding that they currently expend outside the country. While the rationale targeted economic sustainability, it was clearly regressive, and in its focus on the rich, while neglecting the poor, did not fit with the HSSMP focus. The MoH used its commitment to the HSSMP as its benchmark in continued consultations with donors, eventually resulting in a change in the project’s focus and a redesign to support HSSMP strategies. With non-communicable diseases (NCDs) and injuries identified as priority diseases based on the national epidemiological profile, and highlighted in the HSSMP, the focus of the Millennium Challenge Account proposal was reoriented from building a tertiary level diagnostic and treatment center towards combating NCDs and injuries, consistent with the HSSMP priorities. MoH’s persistence in these negotiations was clear evidence of confident ownership of policy directions.

The third key strategic change was the transfer of responsibilities for implementing a SWAp and aid coordination from MoH’s International Cooperation Division to the Strategic Policy and Planning Department, in 2006, a year after the HSACC establishment. This was recognition that aid coordination is not merely a fund raising and reporting task as understood previously, but a strategic function to coordinate, channel and oversee external resources to implement MoH objectives. The changes in the MoH organizational structure that followed the approval of the HSSMP were effectively determined by the need to address functions required to implement key HSSMP strategies. Channeling the domestic and external resources through a better-coordinated strategic framework made off-budget funds more accountable and also created an enabling environment for joint assessments of the performance of the public health sector rather than piece-meal and shielded assessments of the various projects. The trend to more effective coordination has been reflected in the joint sector review of HSSMP mid-term implementation, completed in 2012, using the Joint Assessment of National Health Strategies (JANS) initiated by International Health Partnership( IHP)+ .

#### Challenges

The development of the HSSMP and its implementation processes were not challenge free, and key points have demonstrated the potential vulnerability of the local governance that it has created. With the completion of the HSSMP process, the Core Group was disbanded. The responsibilities of the Core Group are now embodied in the HSACC. The seconded staff now confronted difficulties in returning to their former substantive positions, because of politically driven structural changes in the MoH following the appointment of a new Minister. Despite support for the Paris Principles, some donors have not been comfortable with the level of MoH pressure to re-program their projects to conform with the HSSMP. With persisting ambivalence around MoH capacity, they now considered commitment to the MoH-HSSMP placed implementation of their project resources beyond their control, a risk they were reluctant to take. The high staff turnover and frequent changes in the rules and procedures in the MoH provided some justification for their concerns.

The vulnerability of the key senior MoH staff to a fractious political environment has slowed the pace of implementation of HSSMP and reduced the strength of its influence in aid coordination. The joint sector review of mid-term HSSMP implementation, completed in 2012, also highlighted this loss of momentum in the efforts to accelerate progress towards a SWAp. Senior level staff changes, the infrequency of HSACC operations since 2010 and unclear guidance around implementing a SWAp, were cited as concerns [31]. Clearly, the task of maintaining internal consensus around the HSSMP is critical to extending that leadership to donor coordination.

## Conclusion

Mongolia’s experience shows that the process of developing a national plan, if carried out meticulously, with wide participation and sufficient time for stakeholder consultations, can provide an opportunity to advance ownership, build capacity and lead to better aid coordination in developing countries. The most important success factor for the sustainability of the plan was the commitment of the Government to lead the process, with support from international donors playing a vital role to facilitate this homegrown initiative. Ownership cannot be conferred but can only be claimed [32] and this HSSMP development process has demonstrated that principle.

The strengths of the HSSMP development process lie in three specific areas:

1. ***The continuity provided by political durability, despite political instability***

The HSSMP has “survived” 6 ministers from the time of its development to the current stage of its implementation. This is due to the participatory nature of its development, with consensus building consultative meetings that enabled the HSSMP to consolidate realistic strategies. Strategies to ensure approval by the Cabinet and authorisation by the Prime Minister, development by the staff of the MoH and key stakeholders, and ownership by operational level facilities, have overcome the consequences of the political instability and the staff turnovers that have occurred.

The HSSMP Core Group have ensured continuity and institutional memory remains within the MoH. The choice of a select team of long-term technical advisors maintained continuity and nurtured capacity building in ways that previous short-term technical assistance had not.

2. ***The use of the HSSMP to ensure the cohesion of MoH-led donor alignment***

The MoH used the HSSMP as a clear framework to align partner projects with its priorities and strategies. Donor alignment initiative arose from the strategic plan, rather than being driven by the donors themselves. This was quite a shift, challenging donors’ assumptions about the lack of Government capacity and its commitment to lead donor coordination. The contemporaneous signing of the Paris Declaration by the Mongolian Government, and the development of the HSSMP has had synergies in promoting adherence to the principles of aid effectiveness in the health sector.

3. ***The value of process orientation and participatory approach to building capacity***

The HSSMP development process preferentially used the “learning by doing” approach as a mechanism for creating an enabling environment for increasing MoH ownership and capacity. Participation of domestic and international stakeholders was enabled from the outset and sustained throughout the process through widespread and systematic consultations. The Core Group, embedded in the MoH and consisting of seconded staff, supported the emerging ownership within the Government.

The lessons for Ministries of Health in similar Post-Soviet countries—or other emerging economies where government capacity and local policy processes are relatively strong—are clear: the development of solid governance and technical infrastructure in terms of planning and evaluation provide a solid structure for donor coordination and insure against local political change. The development of a comprehensive policy package—in this case the HSSMP—provides a concrete framework against which donor contributions can be matched. But the governance to maintain this infrastructure is crucial. The disbanding of the Core Group has increased the risk of competing interests within the MoH; the temporary loss of the surveillance provided by the suspension of the HSACC saw unspecified donor funding balloon. Despite the patchy compliance with the Paris Principles [32], donors are sensitive to peer monitoring of their performance; a more structured approach to tracking aid effectiveness through the Paris declaration indicators will enable the gains secured through planning processes to be monitored.

But as important as effective donor coordination is the MoH’s capacity to effectively coordinate its own domestic resources, and to harness the growing contributions of the private not-for-profit and for-profit sector, as well as the emerging public-private partnerships that have resulted from early exploitation of its mineral wealth. As Mongolia’s economic standing increases, and the proportion of donor support decreases, these lessons of coordination will be critical to implementing the MoH’s vision for health.

## Competing interests

The authors declare no competing interests.

## Authors’ contributions

AU conducted the document review, interviews and observation. PH and IN provided the study design and all authors participated in the narrative analysis. AU and AM developed the first draft and led on its further development. PH and IN provided further inputs and revisions throughout the manuscript development process. All authors read and approved the final manuscript.

## Supplementary Material

Additional file 1 **A Total health expenditure and Official Development Assistance (ODA) for health during 2003-2011.** B Main donors in the sector and their contribution. Click here for file

Additional file 2 Roadmap for developing Health Sector Strategic Master Plan. Click here for file
