# Supplementary material for: “In the driver’s seat”: The Health Sector Strategic Master Plan as an instrument for aid coordination in Mongolia
Source: Global Health. 2014 Apr 3;10:23. doi: 10.1186/1744-8603-10-23 (PMC4108099; doi:10.1186/1744-8603-10-23)
Supplement: Additional file 1 — A Total health expenditure and Official Development Assistance (ODA) for health during 2003-2011. B Main donors in the sector and their contribution. [file 1744-8603-10-23-S1.docx]

**Additional file 1:**

1. Total health expenditure and Official Development Assistance (ODA) for health during 2003-2011

| Year | **2003** | **2004** | **2005** | **2006** | **2007** | **2008** | **2009** | **2010** | **2011** |
| --- | --- | --- | --- | --- | --- | --- | --- | --- | --- |
| Health expenditure, total (% of GDP) | 6.56 | 6.24 | 5.27 | 4.78 | 5.27 | 5.82 | 5.79 | 5.51 | 5.26 |
| Health expenditure total (mln US$) | 104.6 | 124.3 | 133.0 | 163.0 | 223.2 | 327.30 | 265.5 | 341.60 | 450.00 |
| ODA for Health-actual disbursed (mln US$) | 6.86 | 4.88 | 6.59 | 5.52 | 16.1 | 13.42 | 13.34 | 25.67 | N/A |

Source: WHO, Global Health Observatory Data in [*http://gamapserverwhoint/gho/interactive_charts/health_financing/atlashtml*](http://gamapserverwhoint/gho/interactive_charts/health_financing/atlashtml)

**Note:** ODA data presented here is not complete as it does not include International NGOs contribution such as ADRA, World Vision and Norwegian Lutheran Mission which are mainly working in regional and district level. Also, different data sources namely, national data bank, WHO and Institute for Health Metrics and Evaluation (IHME) suggest slightly different amounts for ODA. Data collected by individual donors also suggest different (higher) numbers.

Annual government health expenditure has been approximately 250 million USD for the last five year period 2008-2012. Rough estimations based on the data collected by the individual donors suggests that external aid contribution equaled to 10% of the government health expenditure, suggesting the official data on external aid to the health sector is reportedly lower than the actual contribution .

1. Main donors in the sector and their contribution

| Donor | Project focus | Budget year | Amount (USD) |
| --- | --- | --- | --- |
| Asian Development Bank | Health reform in finance and management | 2007-2013 | 16 million |
| World Vision | Child care  Emergency care | 2010-2011 | 2 million |
| UNICEF | Nutrition  Child care | 2010-2011 | 2.5 million |
| GFATM | TB care | 2008-2014 | 10 million |
| GFATM | HIV/AIDS | 2008-2014 | 9 million |
| GFATM | TB-DOTS | 2010-2016 | 9.2 million |
| GFATM | HIV/AIDS high risk group | 2008-2013 | 3.3 million |
| GFATM | National lab network  Blood safety | 2010-2012 | 4 million |
| Australia | Disaster relief  Reproductive health | 2010-2011 | 276,000 |
| Luxembourg | HIV/AIDS prevention in border areas | 2009-2011 | 160,000 |
| UNFPA | Global Program Reproductive Health Commodity Security | 2008-2011 | 3.1 million |
| UN-Trust fund for human security | Human security through Integrated and prevention approaches | 2010-2012 | 319,400 |
| UNFPA | RH service capacity and commodity supply | 2007-2011 | 2.1 million |
| UNFPA | RH quality of care | 2010-2011 | 139, 000 |
| UNFPA & Luxembourg joint project | Telemedicine network | 2007-2010 | 1.5 million |
| WHO | Environmental health | 2010-2011 | 169,500 |
| WHO | New and re-emerging disease | 2010-2011 | 560.000 |
| USAID-Millennium Challenge Account-Health | Prevention and treatment of common disease burden | 2008-2013 | 39 million |
| **Total** | | | **103.3 million USD** |

Source: Ministry of Health, Mongolia, Health sector aid data, 2011

**Note:** This data was collected from individual donors by MoH, Mongolia to capture donor inputs accurately as a part of the aid coordination committee operation. Timeframe varied between the donors as the reporting timeframes of the different donors varied; some donors were only able to provide data covering the last few years. However, this type of data collection is expected to become frequent and a part of the routine data collection to improve aid coordination.
