# Supplementary material for: “In the driver’s seat”: The Health Sector Strategic Master Plan as an instrument for aid coordination in Mongolia
Source: Global Health. 2014 Apr 3;10:23. doi: 10.1186/1744-8603-10-23 (PMC4108099; doi:10.1186/1744-8603-10-23)
Supplement: Additional file 2 — Roadmap for developing Health Sector Strategic Master Plan. [file 1744-8603-10-23-S2.docx]

**Additional file 2**: Roadmap for developing Health Sector Strategic Master Plan
